# Sustainable workforce: South African Audiologists and Speech Therapists

**DOI:** 10.1186/s12960-020-00488-6

**Published:** 2020-07-01

**Authors:** Mershen Pillay, Ritika Tiwari, Harsha Kathard, Usuf Chikte

**Affiliations:** 1 Discipline of Speech-Language Pathology, School of Health Sciences, University of KwaZulu-Natal, Durban, South Africa; 2 Division of Health Systems and Public Health, Department of Global Health, Stellenbosch University, Cape Town, 7505 South Africa; 3 Division of Communication Sciences and Disorders, Department of Health and Rehabilitation Sciences, University of Cape Town, Cape Town, South Africa

## Abstract

**Background:**

Audiologists and Speech Therapists play a vital role in addressing sustainable development goals by supporting people who are marginalised due to communication challenges. The global burden of disease and poor social living conditions impact negatively on the development of healthy communication, therefore requiring the services of Audiologist and Speech therapists. Against this background, we examined the demographic profile and the supply, need and shortfall of Audiologists and Speech Therapists in South Africa.

**Methods:**

The data set was drawn from the Health Professions Council of South Africa (HPCSA) registers (for 2002–2017) for the speech, language and hearing professions. This demographic profile of the professions was created based on the category of health personnel; category of practice, geographical location, population group (race) and sex. The annual supply was estimated from the HPCSA database while the service–target approach was used to estimate need. Additional need based on National Health Insurance Bill was also included. Supply–need gaps were forecast according to three scenarios, which varied according to the future intensity of policy intervention to increase occupancy of training places: ‘best guess’ (no intervention), ‘optimistic’ (feasible intervention), and ‘aspirational’ (significant intervention) scenarios up to 2030.

**Results:**

Most (i.e. 1548, 47.4%) of the professionals are registered as Audiologists and Speech Therapists, followed by 33.5% registered as Speech Therapists and 19.1% registered as Audiologists. Around 88.5% professionals registered as Audiologists and Speech Therapists are practising independently, and 42.6% are practising in the Gauteng province. The profession is comprised majorly of women (94.6%), and in terms of the population groups (race), they are mainly classified as white (59.7%). In 2017, in best guess scenario, there is a supply–need gap of around 2800 professionals. In the absence of any intervention to increase supply capacity, this shortfall will remain same by the year 2030. By contrast, in aspirational scenario, i.e. supply is increased by 300%, the forecasted shortfall for 2030 reduces to 2300 from 2800 professionals.

**Conclusions:**

It is clear that without significant interventions, South Africa is likely to have a critical shortfall of Audiologists and Speech Therapists in 2030. Policy-makers will have to carefully examine issues surrounding the current framework regulating training of these and associated professionals, in order to respond adequately to future requirements.

## Introduction

Health and well-being in South Africa (SA) provide a striking case study of the intersection among economic inequality, social exclusion and human rights deprivations. The Human Resources for Health Strategy and the National Health Insurance (NHI) Bill of 2018 attempts to provide health care to all South Africans within a financial framework to attain universal health coverage and quality health care services. The public sector caters for 84% of the South African population, while 16% of South Africans are served by the private sector. This inequity is further worsened by unequal access and outcomes within and between urban and rural public sector users. Health services, and in particular its human resources component, play an important role in combating inequity and its impact on human rights, human dignity and social justice as it manifests itself on the frontiers of health systems.

The 2030 Agenda for Sustainable Development [1] brings into sharp focus the role of health care professionals with a call to substantially increase health financing and the recruitment, development, training and retention of the health workforce. Within this focus however, what needs to be referenced is health care services to people with disabilities especially those requiring communication interventions. Critically embedded in this problem are considerations of health care human resources. The global burden of illness and disease and social and economic inequalities highlights the lack of services provided to persons with disabilities and their needs. Rehabilitation services, like that provided by Audiologists (Auds) and speech therapists (STs) [also known as Speech-Language Therapists, in South Africa, and Speech-Language Pathologists in other countries like in the United States of America (USA)], are implicated within larger social, political and economic concerns. For example, indigeneity and Western-oriented rehabilitation services concern South African Auds and SLTs similarly to their counterparts in Australia, New Zealand, Canada and the USA. Likewise, accessibility, affordability and appropriate services/technology are all matters configured around equitable provision of services and are global concerns. While these matters are filtered through the distinct lens of being an upper middle-income country in the South, we argue that SA serves as a useful cultural, political and economic case for Auds and STs across high-income and other low-middle income countries (LMICs).

Auds and STs are inter-connected rehabilitation professionals. These professions focus on communication which is critical for human health and well-being. Communication is central to how people socialise, learn and work. Impairments and challenges with communication result in increased co-morbidities such as mental health difficulties making what Auds and STs do critical to any health workforce. The current problem, though, is not only focussing on the need for managing people with communication impairments/disabilities and disadvantage but importantly how we will achieve the goals of a sustainable workforce to deliver such services.

The professional education for speech-language pathology (or ‘therapy’) and audiology in SA began in 1937. The University of the Witwatersrand, Johannesburg, started the first 2-year diploma in Logopaedics in South Africa, which eventually became a 4-year degree in 1946. This single degree qualification provided graduates with the ability to register as both Audiologists and STs. The year 2017 saw the last cohort (from the University of the Witwatersrand) of graduates with this dual registration ability. Currently, all seven universities in SA offer separate professional 4-year degrees that prepare Auds and STs for the workforce. It is a legal requirement that all Auds and STs, meeting the minimum academic training requirements of a 4-year degree, must register to practise with the Health Professions Council of South Africa (HPCSA). The HPCSA registers contain specific demographic data to profile the professions.

Both Auds and STs manage people with communication disabilities that impact on individual’s speech, fluency, language voice and hearing. For Auds, the central practice domain are people with hearing impairments and related auditory system disabilities like balance disorders and tinnitus. STs also manage people with swallowing disabilities. We conducted this study to, firstly, provide a succinct description of who Auds and STs are in South Africa. Secondly, this demographic profile of professionals constituted empirical data to forecast workforce requirements to provide sustainable services to the people of South Africa by the year 2030. Via our case, we hope to raise similar workforce issues relevant for other countries’ consideration.

Hearing loss is among the top three leading causes of years lived with disability (YLDs) at a global level with data from the year 2017 reporting that hearing loss (of > 20 dB) affects 1.33 billion people [2]. Further, the World Health Organization (WHO) estimated that 466 million (6.1%) persons in the world live with disabling hearing loss (defined at > 40 dB for adults and > 30 dB for children) and that by 2050 this will increase to over 900 million people [3]. Significantly, more than 80% of these persons are estimated to be living in LMICs [4] with the biggest burdens being in sub-Saharan Africa and Southern Asia [3]. While it is harder to establish the incidence and prevalence of persons living with swallowing disabilities and/or communication (speech, language, voice and related) disabilities, estimates indicate that 49% of people with disabilities seeking help from rehabilitation services have communication difficulties [5]. The South African national disability prevalence rate is 7.5% [6] with recent data indicating that 1.8% of the population have varying levels of communication difficulties and 3.4% reported hearing difficulties [7]. However, these latter figures are contested due to the data collection methods used (self-reporting vs clinical screening) and severity threshold measures used to estimate prevalence data.

Currently, South Africa has a quadruple burden of illness and disease (BOID). This is the background within which we position hearing, communication and swallowing disabilities, viz. across (i) maternal, newborn and child health illnesses; (ii) human immunodeficiency virus (HIV) and tuberculosis (TB); (iii) chronic, non-communicable disease (cancers, high blood pressure, diabetes); and (iv) the effect of violence and injury in the population. The Institute for Health Metrics and Evaluation (IHME) in 2018 reported that South Africans become disabled and/or die mainly from non-communicable diseases like diabetes and cardiovascular disease, ranked second and third/fourth respectively [8]. Communicable diseases like tuberculosis (ranked highest) and HIV/AIDS ranked in fifth place. Taylor & Ntusi [9] reported on the 2010 SA National Burden of Disease Study which positioned cerebrovascular accident (CVA)/stroke as the ninth most important cause of disability in SA.

The likelihood of South Africa’s BOID being associated with some form of communication (including hearing), balance/vestibular or swallowing disorder is great. For example, survivors of cancer of the head/neck or lungs experience communication and/or swallowing disabilities impacting on their quality of life—all of which remain poorly documented. Similarly, in paediatric populations—especially young children under six, from socially disadvantaged backgrounds, the risk to accessing education and developing adequate language for literacy is a major concern for communication professionals like STs. Children with neurodevelopmental disorders (e.g. cerebral palsy) are likely to have associated eating/drinking difficulties. South Africa has one of the highest HIV/AIDS and TB burdens globally. South Africans with HIV/AIDS or TB and on ototoxic medication live with hearing, cognitive-communication and swallowing disabilities [10]. The burden of disease coupled with social disadvantage which leads to communication, hearing and/or swallowing difficulties is of major public health importance.

Globally, and in rank order, visual, hearing and intellectual disabilities are very common followed by autism spectrum disorder. Of the 52.9 million children from birth to five with disabilities, most (71.3%) live in sub-Saharan Africa [11]. Auds and STs play a critical role in the care of children with these disorders, most of whom have communication (including hearing) disabilities as central to their needs. Many others too—head and neck cancer survivors, people with head injuries and/or trauma and those with pulmonary disorders—may experience hearing disabilities, difficulties in talking/communicating and/or risks of aspiration-related pneumonias due to coping with a swallowing disability.

Historically, the professions of audiology and speech-language therapy have a strong focus on individualised, personal health care [12]; are located mainly in the private sector; and render services mainly to the middle-class urban population [13, 14]. Disparities in disease, access to care and health outcomes vary considerably between and within countries. Indeed, health disparities between urban and rural settings are commonplace in many LMIC in an era of increasing urbanisation. South African rehabilitation services are unevenly provided across its varied socio-economic communities and for its multilingual, multicultural population.

Research into existing human resources capacity in SA is necessary for the planning of health worker training pipelines, the planning of service delivery and the planning of health budgets within the context of goals of the SDGs. An assessment of the current Auds and ST health workforce will assist in the evaluation of shortages, enable the determination of the gaps and provide a basis for forecasting optimal numbers. In order to meet 2030 sustainable development goals, what size of workforce would we need?

This article examines the nature of the Aud and ST workforce in South Africa relative to social and economic contexts to gain insights into their ability to engage in sustainable development and their adequacy in numbers within the health workforce. Additionally, it reports the results of an exercise to estimate the baseline supply of, and need for, Aud and STs in South Africa from 2017 onwards and forecast possible supply–need scenarios up to 2030. The aims of this article are to describe the demographic profile of the workforce regarding public/private sector distribution, population group (race), sex and geographical distribution. We do not aim to provide granular, detailed forecasts but rather to give an overarching view of possible directions of change, in order to inform policymaking.

## Methods

The study was approved by Stellenbosch University’s Health Research Ethics Committee (HREC Reference # X19/06/015). A descriptive, retrospective study design was selected. There is little published, accurate data on the nature of the workforce, its characteristics or variables of interest. A descriptive study design of the workforce is appropriate for the purpose of an exploratory study with the goal of interpreting data toward the ability of the workforce to engage in sustainable development. The Aud and ST workforce was studied in relation to the demands of the 2030 Agenda as developed within the United Nation’s SDGs. Therefore, this retrospective description of the nature of the Aud and ST workforce serves as a useful analytical function to study responses to demands and organisational/workforce change(s).

In context of this study, we need to position what ‘sustainable’ workforce means for the Aud and SLT workforce. Methodologically, this means that, firstly, we need to understand (profile) the past and current workforce toward developing an understanding of what has changed and what needs to change toward the establishment of a sustainable workforce. Embedded in this first goal is the need to position not only basic factors like how many Auds and STs are working in South Africa but also to document if their profile has changed or not. Specifically, this means focussing on characteristics of the workforce such as the categories of workers and their practice (Auds, STs), where they work (geographical location) and related biographical factors that are deemed critical indicators of service provision such as their ages, sex and population group vis-à-vis Apartheid racial classification—all of which are related to South Africa’s political, social, professional and health systems development.

The second critical goal is to then estimate the size of the workforce needed in order to provide appropriate, adequate services to all South Africans by the year 2030.

### Data sources

The main data set for this study was obtained from the registers of the Health Professions Council of South Africa (HPCSA) for speech, language and hearing professions. The HPCSA registers were used to select 12 categories of professionals having qualified as (i) Community Speech and Hearing Workers, (ii) Speech Hearing and Correctionists, (iii) Audiometricians, (iv) Hearing Aid Acousticians, (v) Supplementary Hearing Aid Acousticians, (vi) Supplementary Audiologists, (vii) Supplementary Speech Therapists and Audiologists, (viii) Speech Therapy Assistant, (vix) Speech and Hearing Assistant, (x) Audiologists, (xi) Speech Therapists and (xii) Speech Therapists and Audiologists. Of these categories, student registers are available for Audiologists, Speech Therapists, Speech Therapist and Audiologist and Student Hearing Aid Acousticians. Given their scopes of practice and mandate, we analysed data for persons registered as qualified (i) Audiologists only, (ii) Speech Therapists only and (iii) Speech Therapists and Audiologists (dually registered). Speech Therapists are also known as Speech-Language Therapists in SA (similar to Speech-Language Pathologists in, e.g. North America), hence the use of STs to denote their professional title.

We identified a relatively robust data set period from 2002 to 2017 and accessed all Aud, ST and STA (dual Aud and ST, referred here by the abbreviation Aud-ST) registers from this period for analysis. For the demographic profiling of the workforce, we considered HPCSA data (registrations up to January 2018) (*n* = 3266) to obtain a comprehensive updated demographic landscape of the workforce. However, for estimation purposes, we have considered baseline data up to 2017 (excluding January 2018 registrations) (*n* = 2632).

### Profiling the demographic characteristics of the professions

We adopted the approach taken by Bhayat and Chikte in [15] and collected relevant data using a data collection sheet with the following variables included: (i) category of health personnel; (ii) category of practice, (iii) geographical location, (iv) age, (v) sex and (vi) population group (race) as defined by the South African Population Registration Act (Act No. 30 of 1950) [16] used to classify people as Black, White, Coloured (‘mixed’ ancestory) and Indian (aka South African Indian). Although the Act was repealed in 1991 the use of the categories persists in South Africa in some cases to monitor and evaluate social, economic, political and other forms of transformation. Anonymity and confidentiality of all personnel data were ensured as their names or personal details were neither recorded nor presented.

The public sector figures for Auds and STs (currently employed) across various South African provinces were obtained from PERSAL data available in the 2018 South African Health Review (SAHR) [17]. The difference between these public sector figures from PERSAL and from HPCSA registrations was calculated to obtain an approximate of public-private spilt across the provinces.

This dataset was accessed, collected and analysed by a single operator. The accuracy of the dataset and the analysis was cross-checked by a team member. Data were entered and analysed on a Microsoft Excel spreadsheet and then analysed using the Statistical Package for the Social Sciences (SPSS version 22.0). Frequency distribution, cross-tabulations and graphical representations were used as descriptive statistical methods. Population ratios were calculated, and South Africa’s mid-year population in 2018 was estimated at 57.73 million with an even male to female ratio and a population group (race) analysis indicating 80.8% African, 8.7 % Coloured, 2.5% Indian and 8% White with an even sex distribution across the different categories.

The annual supply of Auds and/or STs was estimated from the HPCSA database. The unique registrations done during the year 2017 under the registry names ‘audiologist’, ‘speech therapist’ and ‘speech therapist and audiologist’ were considered as the supply of trained professionals entering into the South African health system. This number of trained professionals (including community service officers) entering into the workforce in the year 2017 was considered the baseline number for which the supply for 2030 was forecasted. This forecast was predicted using three scenarios of growth in the supply of trained professionals:

- Scenario 1—‘Best Guess’ which worked on the assumption of a 100% growth Auds and STs
- Scenario 2—‘Optimistic’ which calculated professional supply relative to a 200% growth rate
- Scenario 3—‘Aspirational’ which calculated professional supply with a 300% growth rate

The forecasts for Auds and STs were done collectively as there were also registrations under the registry name ‘speech therapist and audiologist’.

### Methodology for estimating the need for Auds and STs

The methodology for need estimation was adopted from similar studies previously undertaken, forecasting requirement of human resources for health (HRH) in the future [18, 19]. Our estimations were calculated using three methods, viz. (i) estimation of need by a service-target approach, (ii) calculation of Aud and ST additional need/posts under the South African NHI system and (iii) Aud and SLT need per million population ratios.

The service target approach identifies targets for production and delivery of the various kinds of health services based on diverse criteria of health need and then convert these targets into the human resources needed to meet them [20].

## Results

### The demographic profile of Auds and STs

**Fig. 1:**
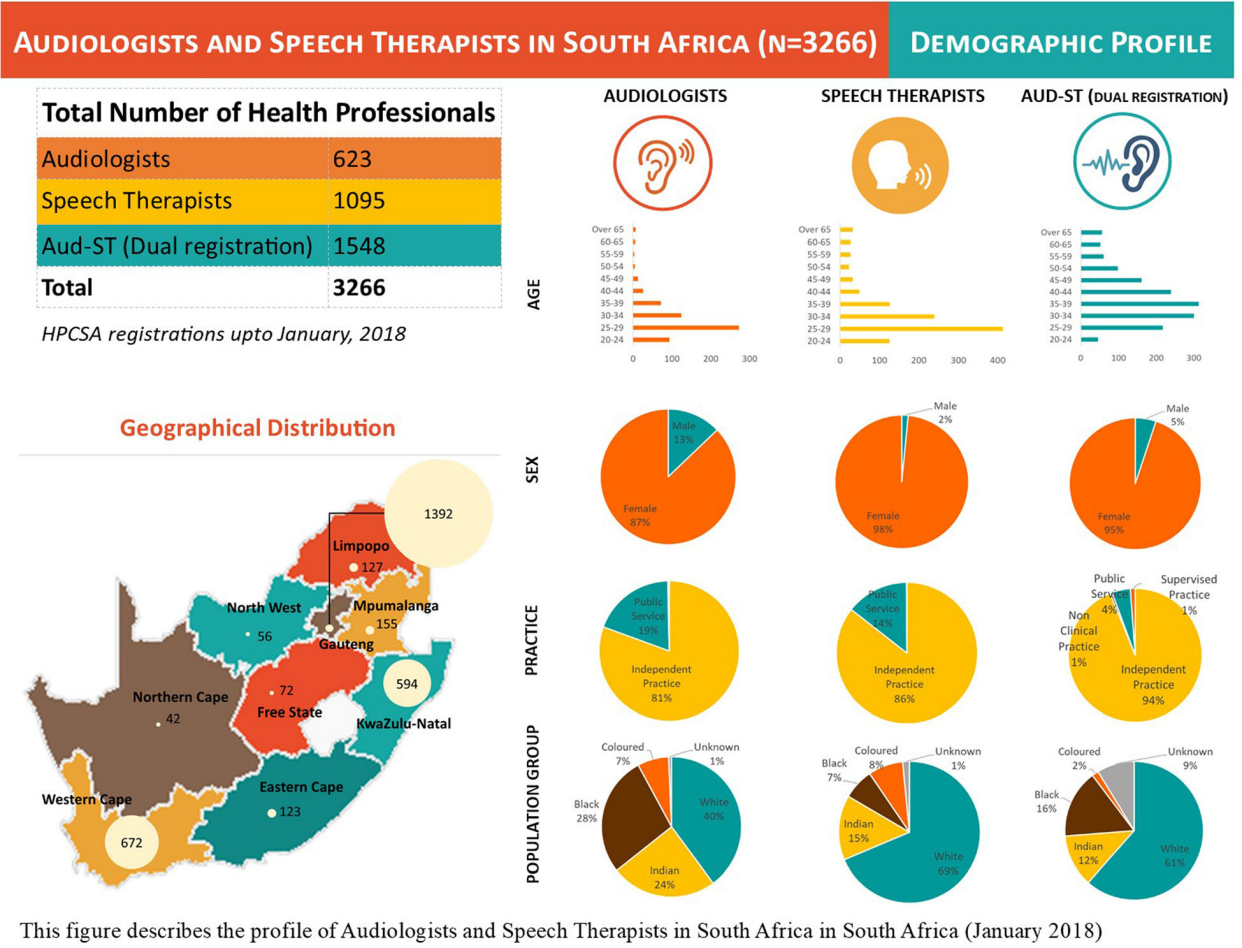
Demographic profile of Audiologists and Speech Therapists in South Africa (*n* = 3266). This figure describes the profile of Audiologists and Speech Therapists in South Africa in South Africa (January 2018)

The HPCSA database had a total number of 3266 registered Audiologists, Speech Therapists and Speech Therapists and Audiologists. Most were registered as Aud-STs (46.8%) followed by STs (33.3%) and Auds (18.9%). The majority of practitioners (94.7%) were females with males comprising 5.3% (Fig. 1). The dominance of females was consistent across different population groups (races). Since 2002, the overall percent of change for males has been higher for the Auds and Aud-STs and lower for the STs.

The four categories of practice include Auds, STs and Aud-STs in (i) independent practice which refers to practitioners who can practice without supervision in the public or private sector; (ii) non-clinical practice, which is a wide category and may include researchers, political appointments and services managers; (iii) public practice signifying that practitioners are restricted to the public sector; and (iv) supervised practice. Notably, most of the workforce (88.5%) are licenced to practice independently in the public or private sector; 10% are restricted to practice in the public sector only with only 1.2% listed in the category of non-clinical or under supervised practice.

Geographical distribution: As displayed in Table 1, the ratio of Aud, ST and Aud-STs is 0.57 per 10 000 population for South Africa as a whole. The Western Cape (1.03), Gauteng ((0.97) and KwaZulu-Natal (0.53) have considerably higher practitioner per 10 000 population ratios than North West (0.15) and Eastern Cape (0.19). The Northern Cape is the largest province (in size) and has the smallest population and thus has a ratio of 0.35.

**Table 1:** Geographical distribution* of Audiologists (Auds), Speech Therapists (STs) and Speech Therapists and Audiologists (Aud-STs) in South African provinces

Category	Audiologist	Speech Therapists	Aud-STs	Total workforce per province	Percentage of total population	Audiologists and Speech Therapists per 10 000 population
Gauteng	184	315	893	1392	25.3	0.97
KwaZulu-Natal	225	239	130	594	19.6	0.53
Mpumalanga	32	38	85	155	7.9	0.35
Western Cape	105	374	193	672	11.5	1.03
Limpopo	17	12	98	127	10.2	0.22
Eastern Cape	31	45	47	123	11.5	0.19
North West	9	13	34	56	6.8	0.15
Free State	8	28	36	72	5.1	0.25
Northern Cape	9	22	11	42	2.1	0.35
Total	620	1086	1527	3233	100	0.57

*Excluding 33 Foreign registered professionals

Age: The age distribution portrays a young profession with 27.6% (902) in the 25–29-year age range followed by a 20.4% (665) cadre aged between 30 and 35 years. A significant 63.6% (2078) of the profession is below 40 years of age while 12.6% (397) are above 50 years of age.

Population Group: Most (59.7%: 1 951) Auds, STs and Aud-STs have identified themselves as White (Table 2). Of the remaining 40.3%, there are 4.7% (156) practitioners who did not identify their population group (race) which matches the same number of people identified as Coloured, the lowest across the population categories. Of the total workforce of Auds, STs and Aud-STs, those categorised as Indians constitute 15.4% (506); persons designated as Blacks comprise 15.2% (497)—almost 50% of whom are Aud-STs. Over the past decade (2008–2017), the overall average annual percentage increment of Auds, STs and Aud-STs has been 9%. The annual percentage increase over the past decade for the different population categories classified as White was 7.9%, for Indians 9.6%, for Coloured 16.1% and those categorised as Black 18.6%.

**Table 2:** Distribution of Audiologists (Auds), Speech Therapists (STs) and Speech Therapists and Audiologists (Aud-STs) by population group (race)

Population Group (race)	Audiologist	Speech Therapists	Aud-STs	Total by across workforce categories (%)
White	248	751	952	1951(59.9)
Indian	152	162	189	503 (15.5)
Black	173	77	247	497 (15.2)
Coloured	45	87	24	156 (4.8)
Unknown	4	17	135	156 (4.8)
Total	623	1095	1548	3266

Sex: Men are in the minority (5%) across the workforce with 80, 17 and 79 (a total of 176) Auds, STs and Aud-STs respectively. Of the remaining 95% or 3090 women workforce, 1469 are Aud-STs, 1095 are STs and 623 are Auds only.

**Fig. 2:**
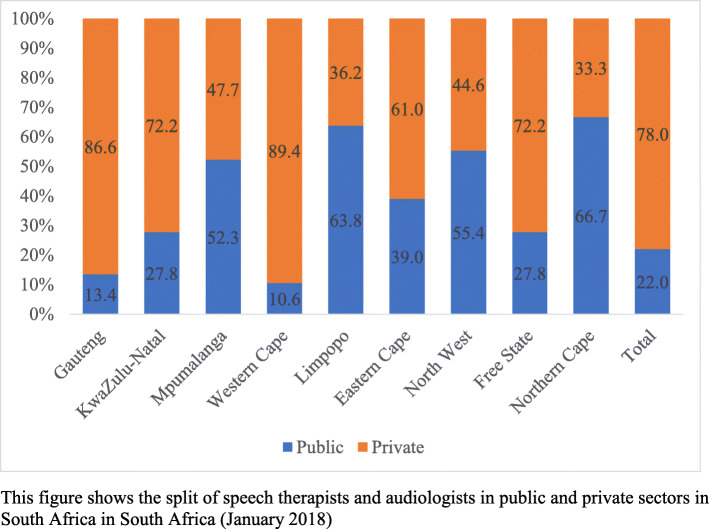
Percentage distribution of Speech Therapists and Audiologists employed in public and private sector. This figure shows the split of Speech Therapists and Audiologists in public and private sectors in South Africa (January 2018)

Distribution of Audiologists and Speech Therapists by sector (public vs private): The numbers of Auds and STs in the public sector have been calculated across provinces as per SAHR 2018 [17] data compared with the provincial geographical distribution of the HPCSA data, illustrating the public-private split for speech therapists and audiologists within South Africa at a provincial level. Practitioners in urbanised and densely populated provinces tend to deliver private services more than public sector services, for instance, Western Cape with only 10.6%, Gauteng at 13.4% and KZN delivering the largest percentage (27.8%) in the public sector. (Fig. 2), The converse is true for less populated provinces like Northern Cape, Limpopo, North West and Mpumalanga with a range of 66.7 to 52.3% professionals in the public sector. Nationally, only 22% of Auds and STs were employed in the public sector.

### Supply and need of Auds, STs and Aud-STs in South Africa

The supply of the Aud and SLT workforce in SA is reported as:

- Supply (baseline) estimated through HPCSA database for 2017.
- Scenario 1 (‘Best Guess’) for a 100% professional supply of Auds and STs.
- Scenario 2 (‘Optimistic’) for a 200% growth rate supply of Auds and STs.
- Scenario 3 (‘Aspirational’) for a 300% growth rate supply of Auds and STs.

Results for Aud and SLT needs in SA are presented as a set of three estimates, viz.:

- Estimated need for Auds and STs by a service target approach.
- Estimate of Aud and SLT additional need/posts under SA’s NHI system.
- Estimates of Auds and STs per million population ratio.

#### Supply estimated through HPCSA database for 2017

As per the unique (new) registrations (including community service officers—CSOs) done in the year 2017 under the HPCSA database, the following numbers were extracted under these registry names (Table 3):

**Table 3:** Supply of Auds and STs in the year 2017 (new registrants as per HPCSA database)

Registry name	Unique registrations in 2017
Audiologists	90
Speech Therapists	98
Audiologists and Speech Therapists	53
Total	241

#### Estimated need for Auds and STs by a service target approach

To illustrate how these calculations were done consider, we offer firstly, how we calculated this need for Auds. The normative need for Auds was calculated from the following estimates [21, 22]. In the health sector, the need for Auds was calculated as follows:

- For primary health clinics (@0.2 × 3475 PHCs) – 695
- For community health centres (@1 × 366 CHCs) – 366
- For a district hospital (@1 × 255 DHs) – 255
- For a regional hospital (@1 × 50 s) – 50
- For a central hospital (@1 × 9 CHs)

Additionally, we calculated:

- For private clinics (@0.2 × 269 private clinics) – 54
- For private hospitals (@1 × 313 hospitals) – 313
- For specialised (public) hospitals (@1 × 90 SHs) – 90

In the research and education (school and higher education), sectors we calculated the need:

- For national research organizations (HSRC/SAMRC) (@5 × 2 institutes) – 10
- For special education schools (@1 × 464 schools) [23],
- For academics at a university (@7^1^ × 7 universities)

We also accounted for Auds needed for the corporate sector, Non-Governmental Organizations (NGOs) and International Non-Governmental Organizations (INGOs) as approximately 100. This provided a total estimated need for 2455 audiologists in 2017. Similarly, the need for STs was estimated to be 2455.

#### Estimate of Aud and SLT additional need/posts under SA’s NHI system

Taking into account the policy imperatives from the Department of Health [24] and based on the implementation of the NHI, there were 76 school mobile units which employed Auds and STs (@1 each). If, for the next 14 years, and assuming that this number is doubled twice in two different phases (e.g. till 2024 and 2030), then the additional need for Aud and SLT posts under SA’s NHI is projected as per the data represented in Table 4.

**Table 4:** Additional need for Auds and STs for NHI posts (2017 to 2030)

Year	Need	Year	Need
2017	152	2024	304
2018	174	2025	347
2019	195	2026	391
2020	217	2027	434
2021	239	2028	478
2022	261	2029	521
2023	282	2030	608

#### Estimates of Auds and STs per million population ratio

For the 2632 registered Aud and SLT workforce registered on the HPCSA database (till 2017), we estimated that 2237 (i.e. 85%) were occupied (in active practice). Thus, the number of Auds and STs per million population based on the 2017 World Bank estimate for the population of South Africa (i.e. 56.7 million) is 39 Auds and 39 STs per million population.

#### Estimates for the Aud and ST workforce gap

**Fig. 3:**
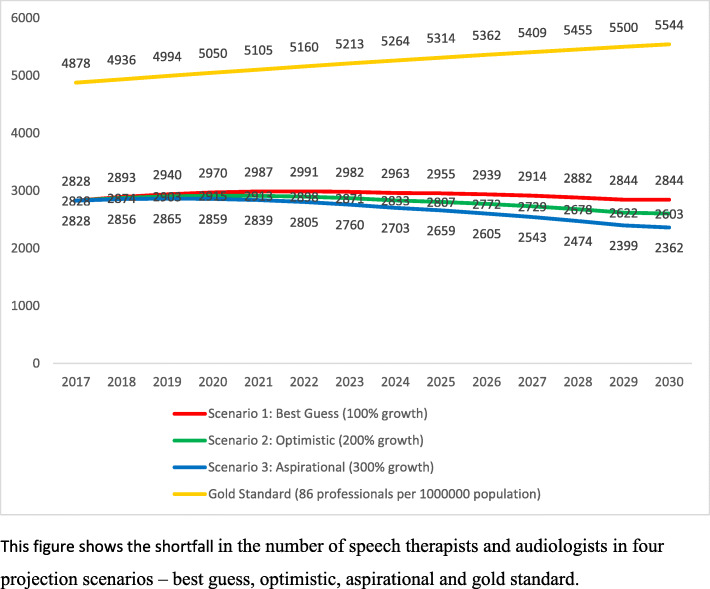
Gap in the number of Auds and STs calculated against a need of 39 professionals per million population with consideration of additional posts under NHI. This figure shows the shortfall in the number of Speech Therapists and Audiologists in four projection scenarios—best guess, optimistic, aspirational and gold standard

In the ‘optimistic’ scenario where we assume that Auds and STs supply are increased by 200% (see Fig. 3) by 2030, then there is a gap of 2000 professionals. If the NHI posts are included in this estimate then, in the year 2026, this number goes up to 2600 professionals.

Based on calculations from data for the year 2017, we recorded the South African population at 56.7 million and 2632 Auds and STs registered with the HPCSA. We assumed that 85% of these professionals are currently employed. Hence, the number of Auds and STs in the health care workforce is 2237. Additionally, as per the unique registrations done under the year 2017, there are 241 professionals joining the workforce. Simultaneously, professionals are exiting the workforce at 9.1 deaths/1000 population [25] and 10% through migration to other countries [26]. This means that approximately 224 professionals are exiting the workforce annually. Thus, net audiology and speech therapy professionals in health workforce (HWF) may be represented and calculated as follows:

$$ {\displaystyle \begin{array}{l}\mathrm{Net}\ \mathrm{Aud}\ \mathrm{and}\ \mathrm{STs}\ \mathrm{in}\ \mathrm{HWF}=\\ {} Number\ of\ Auds\ and\  STs\  in\  HWF+ professionals\ supplied- professionals\ exiting\ workforce\ \left( Optimistic\ Scenario\right)\\ {}i.e.2237+241-244=2234.\end{array}} $$

Further, if these net professionals are deducted from the total need (i.e. 4910 - 2234), then we have an additional need of 2676 Auds and STs. South Africa’s NHI (pilot phase) references the deployment of 76 school mobile clinics [24]. The need has been calculated as 152 (@ 1 Audiologist and 1 Speech Therapist for each school mobile.

Similarly, in the year 2018 for a population of 57.3 million, the number of Auds and STs was assumed to be the net professionals in health workforce in the previous year (2017), i.e. 2234. Here as per the ‘optimistic’ scenario (i.e. Auds and STs’ supply increased by 200% by 2030), 278 professionals are produced. At 9.1 deaths/1000 population and 10% through migration, 244 professionals are exiting the workforce.

$$ {\displaystyle \begin{array}{l}\mathrm{Net}\ \mathrm{Auds}\ \mathrm{and}\ \mathrm{STs}\ \mathrm{in}\ \mathrm{HWF}=\\ {} Number\ of\ professionals\ in\  HWF+ professionals\ supplied- professionals\ exiting\ workforce\ \left(` Optimistic' scenario\right)\\ {}i.e.2234+278-244.\end{array}} $$

Here the normative need has been further forecasted for each year in proportion with an increase in SA’s population until 2030. Thus, if these net professionals are deducted from the total need (i.e. 4969 – 2268), then we derive an additional supply of 2700 Auds and STs. The need for professionals because of the NHI has been accounted as 174 (for the next 14 years where this number is doubled twice in two different phases, e.g., until 2024 and 2030). Thus, the gap is 2800 Auds and STs.

Similarly, in the ‘Best Guess (100% growth) scenario’ in the year 2017, there is a gap of 2800 professionals. However, by the year 2030, this gap will remain as it is, i.e. 2800. In the ‘aspirational’ scenario (300% growth rate) the gap of 2800 professionals reduce to 2300 by the year 2030.

Additionally, we calculated need on the basis of HPCSA service delivery guidelines (HPCSA, n.d.) toward a ‘gold standard’ for Aud and ST workforce (2455 positions each for Auds and STs) for 56.7 million population in 2017. This ‘gold standard’ is calculated at 86 (43 Audiologists & 43 Speech Therapists) per million population. Also, as per the gold standard there existed a gap of 4800 professionals in 2017 which increases to be 5500 professionals till 2030. Thus, Fig. 3 illustrates the four scenarios.

Thus, it is clear that without major workforce planning, South Africa is likely to have a significant shortfall in Audiologists and Speech Therapists in 2030. Policymakers will have to carefully examine issues surrounding the current framework regulating audiology and speech-language pathology education, training and research in order to respond adequately to future requirements.

## Discussion

### Aud and ST workforce and rehabilitation services relative to the South African burden of illness and disease (BOID)

The South African National Health Insurance (NHI) bill implies that Auds and STs who are mainly servicing those who can afford health care (in the private sector) will now be configured into the public sector. However, Aud and ST service provision is particularly complicated by their spatial and geographic distribution. Auds and STs per 10 000 population is 1.03 in the Western Cape, 0.97 in Gauteng, and 0.53 for KZN—with a ratio of 0.35 for both Mpumalanga and the Northern Cape, 0.25 Free State, 0.22 Limpopo 0.19 Eastern Cape and in the 0.15 North West. The 2014 Stats SA survey (based on 2011 data) of persons with disabilities per province revealed that, collectively, the Free State and Northern Cape carry the highest proportion of persons with disabilities at 11%. Conversely, the Western Cape and Gauteng have the lowest percentage of persons with disabilities (5%). As may be seen that there is an inverse relationship between the location of Auds and STs across provinces like Western Cape and Gauteng with the highest proportion of Auds and STs who serve one of the lowest populations (5%) of persons with disabilities, nationally.

Notably, service providers from the racial and linguistic cultural communities constructed under apartheid must also be considered while planning for service delivery within the NHI. The percentages of changes in population groups (races) of Auds and STs have been the greatest for South Africans who are Black (18.6%), followed by Coloured (16.1%), Indian South Africans (9.6%) and White South Africans (7.9%). Approximately one third of the Aud and ST workforce are from the ranks of those who had historically restricted access to these careers and were designated as Black (15.2%), Indians (15.4%) and Coloured (4.7%). There is a mismatch between clients and service providers along racial lines. Significantly, in SA, race implies cultural and linguistic mismatches for the majority of Auds and STs primarily located in the private sector and even for the 22% in public services.

As we transition to an NHI, who will lead us? Who will teach us? Across Auds, STs and Aud-STs, there is an obvious pattern of attrition by age which is possibly due to the relatively low intake/supply of entry into the professions, with the age ranges 25–29 years and then 30–34 years being significantly larger placing the burden of service provision onto a young workforce with experience of as little as 3 to ~ 12 years. Assuming, like other professions, Auds and STs rely on apprenticeships, internships and a (re)growth of the profession via its own institutionalised wisdom, there are few mentors to serve this purpose given the dwindling professional populace aged 45 years and above. This has implications for leadership and academia—besides limiting the workforce to meet service delivery requirements. The arbiters and those with gravitas in the profession are thin on the top of the age pyramid and need to be considered as part of a feed-forward (of supply) concern by graduate professional higher education, considered below.

### Role of mid-level workers

As the nature of service delivery changes in SA, and we develop an increasing awareness for need to develop population-based services, we need to re-look at a workforce that can actually shift services to public services. Firstly, for more equitable distribution of services, we do need more Auds and STs. The Workload Indicators of Staffing Need (WISN) tool used by Department of Health in South Africa arrived at the range of target ratios for Auds at PHC level at 0.05 to 0.1 [27]. In all PHC facilities combined, there are currently 83 STs available in PHCs whereas an additional 324 are needed as per target ratios [27]. The question is will these numbers meet the need?

As per our study calculations, for example under the NHI, it becomes very clear that gap requirements of Aud and STs for 2030 would not be attained with the current workforce supply. Therefore, the role of mid-level healthcare providers should be considered as a key part of service delivery. This cadre of specially trained mid-level providers may provide core services for many underserved populations at primary health centres like PHC clinics. A systematic review undertaken in South Australia highlighted benefits of mid-level workers in the form of improved clinical outcomes, increased patient satisfaction, higher-level services and more time for rehabilitation professionals to concentrate on patients with complex needs [28]. Indeed, in South Africa, persons with disabilities who have access to the services of community rehabilitation workers accessed healthcare services as well as health and education resources better compared to those living in communities without community workers [29].

Thus, we advocate for strengthening the role of mid-level workers which could be a potentially cost-effective and effective way and pragmatic method to increase access to audiology and speech-language pathology/therapy services—both in response to the current rehabilitation needs as well as support the current workforce.

### Impact on Aud and SLT graduate education

Of significance is the historically bounded racial profile of practitioners being educated in SA. Therefore, the challenge to transform practitioners’ racial profile is posted on the gatekeepers’ doors: higher education and the universities. South African new university graduate admission policies together with the establishment of new Aud and SLT programmes (since 2003) at two historically Black universities have led to increased admission of especially Black South Africans. The country’s racialised history that prescribed access (and success) to audiology and speech-language pathology programmes have determined the societies we remain best at serving. Race in South Africa is, undoubtedly, intimately connected to culture, language and several social and economic domains. The politics of race has prescribed audiology and speech-language pathology/therapy services whose traditional beneficiaries are heteronormative Anglo-Saxon, Judeo-Christian cultures. Therefore, the numbers, percentages and other data we provide in this paper cannot be decontextualized from the cultural, linguistic, economic and even geographic (urban/rural) orientations of these cultural capitals. Clearly, what our data demonstrate is that although discriminatory legislation that shaped the racial profile of the profession has been abolished, racial inequities continue to haunt the profession in terms of its paucity of Black and Coloured South Africans.

While race is a critical indicator to evaluate SA’s transformation in higher education, a blind spot is the female to male ratio. It is by no accident that, soaked in a heteronormative society where women are constructed as carers, the professions persist in maintaining a dominant (95%) female workforce. Additionally, the grander, globalised shape of health care that elevates individual, personal health care models as the ‘gold standard’ contextualises why more practitioners are in the private sector. In summary, key indicators like race, gender and location of services all indicate that higher education and graduate development remains challenged by several intersecting points in dire need of transformation.

### Need for undertaking further HRH planning and forecasting for Aud and STs

There is a need to develop clear staffing norms (possible benchmarks) toward ensuring equity in HRH distribution of Auds and STs. Thus, specific staffing norms will lead to specific service targets for specific health outcome targets in rehabilitation. It is suggested that norms should be developed in ranges (minimum-maximum as per various scenarios, above) that may provide some room for flexibility in the system.

For Auds and STs and other rehabilitation practitioners, an estimated gap approach that considers needs will be better suited to SA, also indicated in the Percept report on medical specialist planning in South Africa [30]. In 2019, Rispel et. al indicated that some of the components of an effective HRH planning and forecasting model include (i) demographic and epidemiological changes, (ii) impact of health policies on service delivery, (iii) quality and equity, (iv) prioritisation of underserved areas, (v) workforce and health expenditure, (vi) level of services and (vii) the productivity of healthcare workers [31].

Thus, in a resource-constrained country like South Africa, there is a need for undertaking a granular HRH planning and forecasting exercise. For rehabilitation services like audiology and speech-language pathology, this necessarily means greater focus on substantial elements such as disease burden, current fiscal space, existing geo-spatial and social inequities to access to rehabilitation services.

### Critique of the study

Many aspects regarding the audiology and speech-language pathology/therapy services and of its workforce in South Africa are unknown. Therefore, we developed a series of assumptions and a predetermined, but limited, set of influencing factors when designing the study. Assumptions adopted were similar to previously undertaken HRH forecasting studies [18, 19]. For example, workforce attrition was rationalised at two levels only, i.e. death and migration. However, other reasons such as change of profession or retirement were not included. We made no attempt to adjust for the different types or duration of the professional education programmes for Auds and STs. We have also not accounted for the possibility of individuals working in more than one discipline. Changes in technology (increasing efficiency) of current workforce were also not considered for the projections.

HPCSA registers do not capture data regarding the employment sector where their health professionals work. The inequity in access between the public and private sectors remains unreported [31]. Also, HPCSA registers do not provide reports on emigration, death and retirement of health professionals or those who are registered but inactive [31]. Other ‘missing’ data, needed to contextualise such a workforce study, include details about the HRH primary practice site (private/public), current job held and nature of employment status (full/part-time).

## Conclusion

In summary, there are a total of 3266 registered Auds and/or STs and 94.7% are female. Over time, STs have maintained their sex profile more than Auds or Aud-STs. The practitioner to population ratio is 0.57 per 10 000 population, with the Western Cape (1.03), Gauteng ((0.97) and KwaZulu-Natal (0.53) faring better than the North West (0.15) and Eastern Cape (0.19) provinces while the Northern Cape (the largest province) has a ratio of 0.35. The professions are young with 27.6% aged between 25–29 years and 20.4% are 30–35 years. 63.6% are below 40 years of age while 12.6% are above 50 years. While 4.7% practitioners did not identify their population group (race), 15.4% identified as South African Indians, with Black and Coloured at 15.2% and 4.7% respectively. White practitioners dominate at 59.7%. Overall, only 22% of the workforce are employed in the public sector.

The total estimated need calculated across practice domains was 4910 with 2455 each for Auds and STs. When accounting for the NHI, the additional need was calculated with a range from 152 to 608 (for 2017– 2030). Estimates were calculated at 39 Auds and 39 STs per million population, while the workforce gap was estimated across four scenarios. Based on the NHI, the need was calculated at 174 over 14 years with a gap of 2800 Auds and STs. In the ‘Best Guess (100% growth) scenario’ in the year 2017, there is a gap of 2800 professionals which remains as it is, i.e. 2800 by the year 2030. In the ‘aspirational’ scenario (300% growth rate), the gap of 2800 professionals reduces to 2300 by the year 2030. A ‘gold standard’ for Aud and ST workforce was calculated at 86 (43 Auds and 43 STs) per million population.

In conclusion, the case of audiology and speech-language pathology services provides insight into the HRH crises in South Africa. Our huge BOID and associated communication (including hearing/related system) and/or swallowing disabilities must be managed with due diligence. Valid and reliable estimates, viz. compiling information about supply and need, demands and supply aspects, are a dire need. In this paper, our intention was not to provide granular, detailed forecasts but rather an overarching view of possible directions of transformation to inform policymakers.

## Data Availability

Yes

## Abbreviations

Aud: Audiologist
Aud-STs: Audiologists and Speech Therapists
BOID: Burden of illness and disease
CH: Central hospital
CHC: Community health centre
CSO: Community service officer
CVA: Cerebrovascular accident
DH: District hospital
HIV/AIDS: Human immunodeficiency virus infection and acquired immune deficiency syndrome
HPCSA: Health Professions Council of South Africa
HREC: Health Research Ethics Committee
HRH: Human resources for health
HSRC: Human Sciences Research Council
HWF: Health workforce
IHME: Institute for Health Metrics and Evaluation
INGO: International Non-Governmental Organization
KZN: KwaZulu-Natal
LMIC: Low-middle-income countries
NGO: Non-Governmental Organization
NHI: National Health Insurance
PERSAL: Personnel and Salary System
PHC: Primary Health Care
RH: Regional hospital
SA: South Africa
SAHR: South African Health Review
SAMRC: South African Medical Research Council
SDG: Sustainable Development Goals
SLT: Speech Language Therapist
ST: Speech Therapist
TB: Tuberculosis
USA: United States of America
WHO: World Health Organization
WISN: Workload Indicators of Staffing Need
YLDs: Years lived with disability

## Footnotes

1 Minimum required number for UG programmes has been considered here
